# Scales of Cancer Evolution: Selfish Genome or Cooperating Cells?

**DOI:** 10.3390/cancers14133253

**Published:** 2022-07-01

**Authors:** Branislav Brutovský

**Affiliations:** Department of Biophysics, Faculty of Science, P. J. Šafárik University, Jesenná 5, 041 54 Košice, Slovakia; branislav.brutovsky@upjs.sk

**Keywords:** intratumour heterogeneity, phenotypic switching, bet hedging in cancer

## Abstract

**Simple Summary:**

Cancer continuously evolves its ability to survive in time-varying microenvironment, which results, regarding the therapeutic context, in its therapeutic resistance. As it is accepted that the development of resistance is the direct consequence of intratumour heterogeneity, its evolutionary etiology is intensively studied. Models of carinogenesis are often assessed accordingly to how well they fit into the evolutionary scenario. In the paper, the relevant observations and concepts in cancer research, such as intratumour heterogeneity, cell plasticity, and Markov cell state dynamics, are reviewed and integrated into an evolutionary model. The possibility that the interaction between cancer cells can be interpreted as cooperation is proposed.

**Abstract:**

The exploitation of the evolutionary modus operandi of cancer to steer its progression towards drug sensitive cancer cells is a challenging research topic. Integrating evolutionary principles into cancer therapy requires properly identified selection level, the relevant timescale, and the respective fitness of the principal selection unit on that timescale. Interpretation of some features of cancer progression, such as increased heterogeneity of isogenic cancer cells, is difficult from the most straightforward evolutionary view with the cancer cell as the principal selection unit. In the paper, the relation between the two levels of intratumour heterogeneity, genetic, due to genetic instability, and non-genetic, due to phenotypic plasticity, is reviewed and the evolutionary role of the latter is outlined. In analogy to the evolutionary optimization in a changing environment, the cell state dynamics in cancer clones are interpreted as the risk diversifying strategy bet hedging, optimizing the balance between the exploitation and exploration of the cell state space.

## 1. Introduction

**Figure 1:**
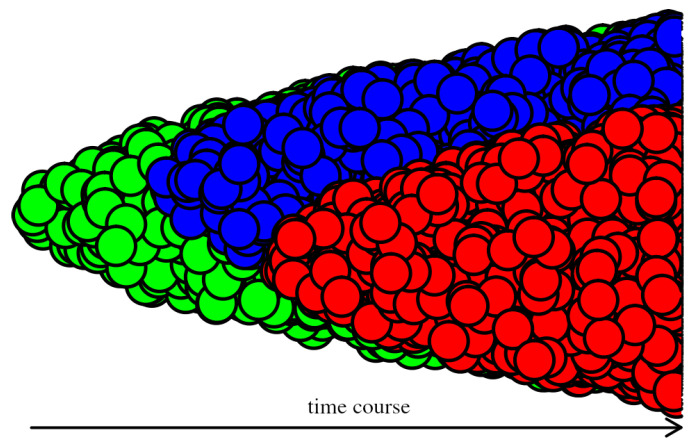
Simplified model of cancer evolution. The new clone originates by mutation(s) from the original clone.

Nowadays, evolutionary theory is broadly accepted as an instructive conceptualization to understand the basic etiology of cancer at an intuitive level [1,2,3]. In simple metaphorical scenarios, the cells ‘compete’ for resources to maximize their respective offspring. After many reproduction loops, some of the cells acquire mutations that increase their proximate reproductive fitness by acquiring, step by step, specific phenotypes characterized by self-sufficiency in growth signals, insensitivity to growth inhibiting signals, evasion of apoptosis, limitless replicative potential, sustained angiogenesis, and tissue invasion and metastasis, together termed as the ‘hallmarks of cancer’ [4]. The clones expanded by these ‘lucky’ cells consequently prevail in the population (Figure 1). Although basic evolutionary principles are already reflected in cancer treatment (as exemplified by combination therapies and drug holidays), recognition of more advanced evolutionary features in cancer progression and addressing them by the therapy could bring better perspectives for patients [5,6,7,8]. Gatenby et al. showed that the evolutionary motivated adaptive therapy may provide substantially longer survival than the standard high dose density strategies [9]. In the strategy of benign cell boosters, Maley and Forrest proposed to increase intentionally proliferation rate of the benign cells sensitive to cytotoxin and then apply the toxin [10]. In the strategy design by Chen et al. [11], the ’evolutionary trap’ selects from a karyotypically divergent population the subpopulation with a predictably drugable karyotypic feature. The evolutionary double bind strategy to control cancer by Gatenby et al. [12] exploits the fact that therapy resistance requires costly phenotypic adaptation that reduces the fitness of the respective cells [12]. Recently, it has been shown that the proliferation of malignant cells can be decreased by the administration of non (or minimally) cytotoxic ersatzdroges [13,14] thereby the cell’s resources are diverted from the proliferation and invasion towards the efflux pump activity. During recent years, directed evolution of oncolytic viruses with desirable properties has been investigated in the virotherapy [15].

Despite many years since postulating the ’substrate-free’ principles of evolution by Darwin [16], a lot of universal questions in the theory of evolution is still open. Evolution was conceptualized as an abstract search process through the astronomically huge search space of combinations of genes a long time ago [17]. Quantifying the quality of each combination of genes by its fitness value, the evolution was identified with a search for the highest peak (maximum fitness) in the abstract fitness landscape [17]. In this way, evolution has been linked with the field of optimization as conceived in engineering and economy [18], initiating the new branch of stochastic optimization techniques, presently known as evolutionary algorithms [19]. Despite the fact that, unlike biological evolution, in evolutionary optimization the fitness function is purposefully constructed, success or failure of either process depends on universal mathematical properties of the fitness landscape. Being applied across the wide range of applications, as diverse as optimization, sociology, or ecology, evolutionary algorithms can potentially predict many universal features in the behaviour of evolving populations, and it is universal enough to be applied to design novel, evolutionarily motivated, anticancer therapies.

A few recent observations in cancer biology and therapy, such as significantly increased non-genetic intratumour heterogeneity, therapy-triggered phenotype switching [20], or Markov property of cancer cell state dynamics [21] challenge the evolutionary concept of cancer progression and its relevance for the therapy design. Respecting Theodozius Dobzhansky’s statement *’Nothing in biology makes sense except in the light of evolution’* [22], we review the evolutionary scenario for cancer in attempt to integrate the above observations in it. Cancer is conceived as the optimization process in time-varying fitness landscape and non-genetic intratumour heterogeneity as the implementation of risk-diversifying strategy preventing the cancer cells’ extinction in uncertain or changing tumour microenvironments.

## 2. Biological Background

### 2.1. Intratumour Heterogeneity

Many advanced tumours have poor clinical outcome due to developing resistance to therapy, which is caused by extremely diverse mechanisms [23]. Selection for in vitro drug resistance can result in a complex phenotype with more than one mechanism of resistance emerging concurrently or sequentially [24]. Genetic data shows that tumours contain complex combinations of low frequency mutations providing different cancer phenotypes [25,26,27,28]. It was demonstrated that accumulation of viable clonal genetic variants poses greater threat of progressing to cancer than homogenizing clonal expansion [29,30]. It was reported that genotoxic stress induces several cell death pathways, not all satisfying classical definition of apoptosis [31]. Previous studies indicate that possibly no prototypical cancer genotype exists and every tumour carries a unique set of mutations, indicating that multiple genetic pathways may lead to invasive cancer as would be expected in a stochastic non-linear dynamical system [32,33].

It is intuitively accepted that experimentally observed intratumour heterogeneity (ITH) increases the probability that the therapy resistant clones appear. The same is true for the immune response, where a huge number of alternations in the genomes of cancer cells results in an altered repertoire of antigens, thereby cancer cells loose their immunogenicity. Presently, ITH is viewed as the central obstacle in the therapy design, and understanding its causes, structure and dynamics poses challenge to cancer therapy [34,35,36,37,38,39,40]. If no similar enough pattern in ITH exists and each tumour contains unique, nevertheless causative set of mutations, the therapy design trying to address cancer’s weak point by generalizing the data from many samples could be fruitless.

Apart from genetic heterogeneity, cancer cell populations show huge non-genetic phenotypic heterogeneity due to epigenetics, which becomes a challenging research topic [41]. Many authors propose that the genetic heterogeneity is unlikely to be the major contributor to phenotypic heterogeneity in general, but, underlying heritable differences, fuel tumour evolution [42,43]. It has been known for a long time that epigenetic changes, such as DNA methylation, histone modifications, chromatin remodeling, and small RNA molecules, play a causative role in cancer initiation, progression, and resistance [44,45,46,47]. It has been reported that epigenetic defects, such as promoter CpG island hypermethylation-associated silencing of DNA repair genes, are known to cause genetic changes, and translocations and mutations can cause epigenetic disruption [48], which means that, on the one hand, mutations in epigenetic regulators lead to an altered transcriptome, and, on the other hand, epigenetic silencing of DNA repair genes is responsible for genetic instability in cancer cells [49].

Genetic mutations and epigenetic changes fundamentally differ in their respective characteristic timescales. Regarding short timescales of epigenetic changes [50], it is clear that non-genetic heterogeneity dominates in the rapid, spontaneous phenotypic diversification of a clonal (isogenic) cell population within a homogeneous environment [43] and, consequently, the therapeutic resilience should be attributed not only to the genetic diversity but to epigenetic plasticity as well [51]. Non-genetic heterogeneity can contribute to the somatic evolution of cancer cells by accelerating tumour progression and development [51,52,53,54].

### 2.2. Phenotypic Plasticity

**Figure 2:**
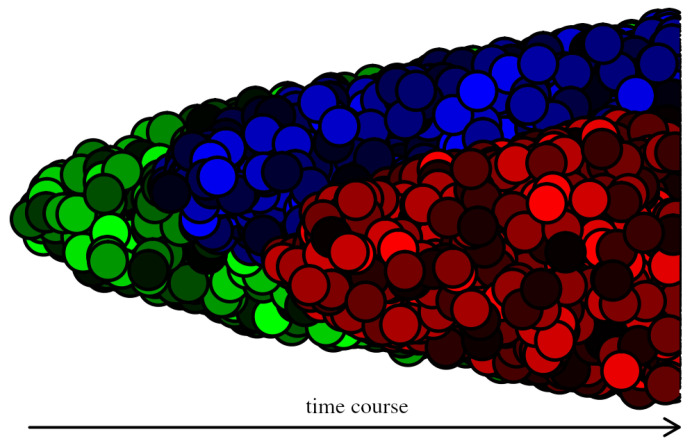
Three genetically different (cancer) clones are depicted—green, blue, and red. In difference with Figure 1, each clone consists of cancer cells in different states, plotted with different shades.

In evolutionary biology, the term ’phenotype’ refers to the set of observable characteristics of an individuum caused by the genotype, which forms a stable end-state on which selection acts. For a long time, the phenotypic heterogeneity of cancer cells was attributed exclusively to genetic variability, implicitly adhering to the one-to-one genotype-phenotype mapping (Figure 1). Presently, it is broadly accepted that non-genetic mechanisms, such as gene expression noise and multiplicity of stable states in gene networks, are responsible for the phenotypic identities of normal cells [42]. It implies that these mechanisms contribute to the phenotypic heterogeneity of cancer cells as well [55], which is supported by many papers that show that populations of isogenic cancer cells may consist of phenotypically different subpopulations [56,57,58,59,60,61] (Figure 2). The ability of the genotype to produce more than one phenotype is called phenotypic plasticity. If the phenotype changes without a genetic cause, it is termed the phenotypic switching.

Presently, phenotypic plasticity is attracting much attention, as the intraclonal variability in phenotypic characteristics of isogenic cancer cells affects cancer progression and its response to treatment [62,63,64]. Evidence of cancer cell plasticity motivates the effort to stimulate (or prevent) specific phenotype switching purposefully as a therapeutic strategy [65,66,67]. However, it requires not only deep knowledge of molecular mechanisms responsible for the cancer cell plasticity [68], but understanding its dynamics at a conceptual level as well.

Intratumour heterogeneity and phenotypic plasticity typically lead to the resistance to therapy, including targeted therapies, which starts at the same time as the therapy and crucially decreases its efficiency [38,64,69,70,71,72,73,74]. The reason is the static fashion of most typical therapies [14], which contrasts with the adaptive nature of resistance. Adaptivity of the immune system can be viewed as a motivation for the novel therapeutic approaches. Recently, the conceptual paper conceiving therapy as an evolutionary process was published [75].

### 2.3. Cancer Cell State Dynamics

To express cell properties that result from the interplay of the genome, epigenome, transcriptome, and proteome, the abstract term ‘cell state’ is often used instead of the terms cell type or phenotype [76]. Due to their tendency to be self-stabilizing, there are typically fewer distinct cell states in a tumour than it could be inferred from the degree of genetic, epigenetic, and transcriptional heterogeneity, and genetically distinct cells may be susceptible to treatment with the same drugs [77]. Distinguishing between phenotypic differences caused by the changes in their DNA sequences and those resulting from the epigenetic modifications is, nevertheless, instructive for the biological insight as well as for the therapy design.

**Figure 3:**
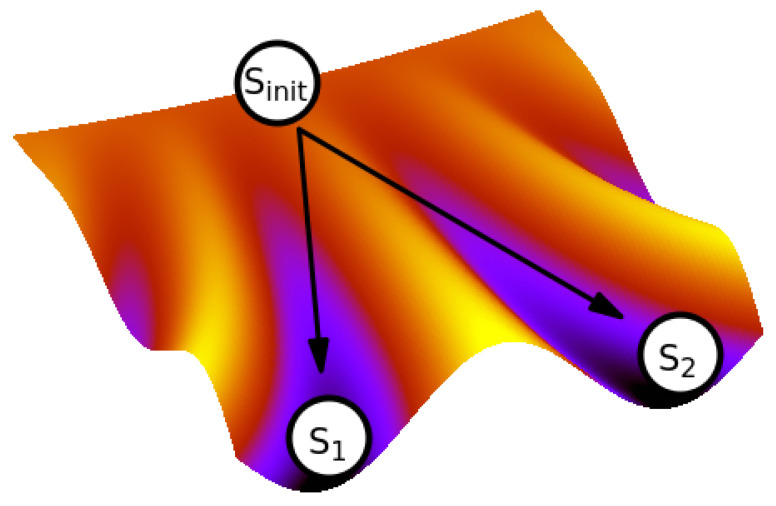
The epigenetic landscape. The cell state is represented by a ball rolling down from an undifferentiated initial state $S_{\mathrm{init}}$ along one of the ‘canals’ until it reaches the respective differentiated state (here $S_{1}$ or $S_{2}$).

The relation between genetics and epigenetics was formulated by Conrad Waddington long time ago. He conceptualized the process of cellular differentiation as the movement of the cell state in the ‘epigenetic landscape’ [78] (Figure 3), where each genome provides a unique landscape that assigns stabilities to all the genome’s cell states. The landscape contains the areas of stable states, epitomizing the cell types, to which the cells, initially undifferentiated, are ‘canalized’.

Despite Waddington’s conceptualization, which is often reduced to the visualization of a simplified differentiation process that can diminish its explanatory power [79], it enables to illustrate the difference between the cell state commitment of normal and cancer cells in an instructive way [43]. Therein, the epigenetic landscape, changed either due to genome mutation(s) or microenvironmental change can provide a new repertoire of the stable cell states (‘attractors’), also in the areas not occupied in the original epigenetic landscape. However, as these do not undergo selection process, they are not evolutionarily harmonized with the needs of the tissue and become, from the viewpoint of multicellular organism, pathological [43].

It was observed that isogenic cancer cells actually switch between different cell states in a reversible way [56,57,58,59,60,61], challenging the concept of cancer cells stemness. Phenotypic plasticity enables cancer cells to shift dynamically between a differentiated state with limited tumourigenic potential and an undifferentiated (stem-like) state [68], to escape a targeted therapy by switching to an alternative phenotype [20,70,74,80], to switch between the proliferative and invasive phenotypes defined by the high and low expression levels of microphthalmia-associated transcription factor [81,82], or switch reversible between drug-sensitive and drug-tolerant phenotypes [57]. Similarly, melanoma progression to an invasive state, linked to an increase in copy number alterations, suggests that changes in gene expression (hence transcriptional programmes) are involved in this process [83,84]. Many forms and mechanisms of phenotypic switching were observed at molecular, genetic and expression levels [85,86,87,88,89] as well as theoretically studied [90,91,92,93,94,95].

In Ref. [21], the authors studied phenotypically structured population of human breast cancer cells, consisting of three fractions of cells in different states (stem, basal, and luminal). By studying the cell state dynamics of the fractions, they found that these remain, under stationary conditions, in equilibrium proportions [21]. Moreover, if the cancer cells population was purified for any of the three cell types, the equilibrium rapidly re-established [21]. As the growth towards equilibrium proportions would require implausibly high proliferation rates, the authors concluded that the progression towards equilibrium was not due to differential growth rates of the respective phenotypic fractions, but rather to the stochastic transitions between the three cell states [21]. Summarizing their observation, they suggested that the observed cell state dynamics is Markov process, which implies that the three phenotypic fractions correspond to the stationary distribution of the cell state dynamics with constant (under fixed genetic and environmental conditions) interconversion rates [21]. In this way, stochasticity at the single cell level is linked with the determinism at the cell population level. Despite that the fate of the individual cells is uncertain, the cell state abundances are fixed, which enables to maintain the tissue’s biological function [96].

Cancer research concentrates predominantly on the molecular aspects behind the cell states transitions. Despite the physical processes behind the respective elements of the transition matrix being constrained and depending on each other, the huge number of degrees of freedom still leaves opportunity to get a plethora of stationary distributions by multiple combinations of interconversion rates (and the respective physical processes), which is consistent with many forms and mechanisms of phenotypic switching observed at molecular, genetic, and expression levels [85,86,87,88,89]. On the other hand, the Markov model enables to study universal mathematical properties of the cell state dynamics separately from its molecular basis, such as the convergence of the distribution of the cell states towards stationary distribution, which is fully determined by its underlying probabilistic transition matrix. Recognizing that equilibrium cell state abundances do not depend on the initial cell types proportions themselves but rather on the transition probabilities, which is the fundamental property of Markov processes, can have far reaching consequences to the therapies that target cells in specific states, such as stem cells [21]. It implies that the composition of the cancer cell population can be changed not necessarily by targeting the cells in the specific cell state(s), but, eventually, by modifying one or more transition probabilities instead.

## 3. Evolutionary Aspects

As biological organisms were, for a long time, the only ’experimental’ system to study evolution, the evolutionary rules fused, step by step, with the implementation details, shifting the concept of ’gene’ from its original meaning, which is the cause of an inheritable phenotype characteristics, to its present meaning, as the specific physical structure (the part of the DNA) [97]. This shift has motivated cancer researchers to concentrate more on the molecular (or physical) aspects of cancer, such as genetics and epigenetics, than on universal evolutionary rules, which implicitly refer to the specific selection level and timescale. To incorporate phenotypic plasticity into evolutionary cancer etiology, firstly we reconsider fundamental evolutionary issues, such as the cancer relevant-selection unit, the timescale of cancer progression, and the fitness of the selection unit on that timescale.

### 3.1. Cancer Relevant Scales

Selection level is in evolutionary biology frequently discussed topic [98]. The wild type genome in metazoan comes as an evolutionary compromise between (at least) two selection levels—cellular (individual) and multicellular (group), each with its own selection unit (cell and organism, respectively), characteristic timescale (an ‘investment horizon’), and parameters and purposeful definitions of fitness. Selection pressure to intercellular interaction between the cells within normal multicellular body comes from a higher than cellular level, which typically decreases the (reproductive) fitness of the selection unit at a cellular selection level [99]. As a consequence, normal somatic cells within metazoans do not evolve during the individual’s life and their homeostasis is maintained by the tissue signals and internal controls [100]. In contrast to normal cells, cancer cells do evolve, owing to mutations enabling them to bypass the mechanisms that restrain proliferation and the function and identity of the normal metazoan cells [101]. As this ‘short term’ evolution, during the organism’s lifespan, is biased towards maximizing the cellular fitness at the expense of the organismal fitness, cancer cells are often termed ‘selfish’.

The phenotype represents collection of all features of the selection unit, which derive from the genotype and undergo selection. In the evolutionary causation of cancer, cancerous features (‘cancer phenotype’) are usually attributed to the cell. Consequently, the cell is, owing to well understood cellular mechanisms of replication and inheritability, intuitively assumed the basic cancer-relevant selection unit with the fitness derived from its proximate reproduction capability. Observed phenotypic plasticity of isogenic cancer cells complicates the above simplified evolutionary scenario, as different phenotypes confer, in general, different fitness to the isogenic cells. This, together with the inherent uncertainty of a tumour microenvironment, makes the cell’s fitness stochastic quantity and the role of Darwinian evolution in cancer progression less convincing (or, at least, less obvious) [102,103].

In principle, both cancer cell as well as cancer clone, satisfy the three basic pillars of evolution theory [16] and represent two, rather parallel than alternative, selection units. The evolution of each can be studied on its characteristic timescale, with the respective phenotype and fitness. This ambiguity implies the fundamental question: Which selection unit dominates in cancer progression?

To answer the question I suggest that the only cancer-relevant selection unit is the genome, and the cell and clone are viewed as the genome’s phenotypes at two different timescales conferring them their respective fitness. Intuitively, the higher the selection level is, the longer timescale is needed to reflect the fitness of the respective selection unit, which implies the discussion regarding the most appropriate timescale to refer the fitness of the cancer genome. For instance, at the proximate timescale, such as the cell’s doubling time, the fitness of the genome reflects the cell’s ability to undergo division. At a longer timescale, after the original genome produces a clone, the fitness of the genome intuitively derives from its clone’s size. Obviously, the evolutionary outcome of the genome at short timescale does not necessarily correlate with its evolutionary success at a longer timescale. Studying the short- and long-term evolutionary success in general case, Palmer and Feldman suggested two metrics, *k*-fitness and *k*-survivability [104]. The former quantifies the probability of increase of the size of the respective lineage after *k* generations, the latter relates to the likelihood that the species will avoid extinction after *k* generations. Intuitively, if *k* increases, *k*-fitness of the genome depends more and more on the eventual interaction of the cells in the clone.

Taking into account the time that cancer needs to progress sufficiently to impact human health and/or an eventual therapy outcome, the long-term evolution of cancer genome is more relevant than its short-term evolution. Assuming the longer timescale of cancer progression, the more complex phenotype responsible for the genome’s fitness can be considered and less intuitive (nevertheless more efficient) strategies to maximize it can the genome evolve.

### 3.2. Cancer Genome’s Fitness

**Figure 4:**
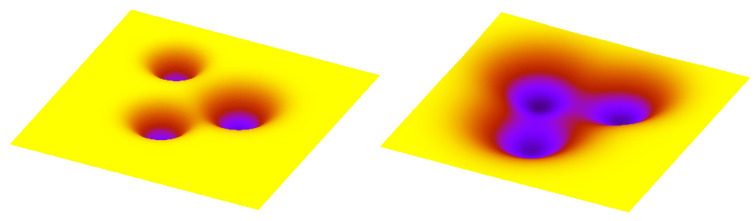
Transition probabilities can be instructively visualised by the heights of the barriers which separate areas corresponding to stable cell types. Higher barriers correspond to more restrictive (i.e., less plastic) epigenetic landscape (**left panel**), while the lower barriers (**right panel**) correspond to more permissive landscape.

Before the evolutionary modus operandi of cancer can be successfully addressed by therapy, the crucial questions concerning the cancer genome’s fitness must be understood: (i) what are the parameters of the genome’s fitness, (ii) what is the optimum genome’s fitness, and (iii) why the cancer genome outperforms normal genome in the fitness defined by these parameters. Assuming the longer timescale of cancer progression, the genome’s fitness can be separated from the reproductive fitness of the cell in which the genome resides, and include the intercellular interaction between the cells within the isogenic clone. Having conceptualized basic biological mechanisms behind the non-genetic heterogeneity, such as Waddington’s epigenetic landscape and Markov model of the cell state dynamics, the genotypic space can be identified with the search space and the cell state dynamics with the fitness evaluation process. As a result of the cell state dynamics, the genome’s clone consists of a few different, mutually interacting cell state abundances, each with its respective cell’s replication and survival probabilities. Straightforwardly, as isogenic cells in different states differ in their properties, the genome’s fitness is, on cancer relevant scales, determined by the sizes of the cell state abundances. These correspond to the rates of interconversions between states [21], which are underpinned by the respective physical processes. In this way, the genetically coded molecular mechanisms are under evolutionary pressure, which selects those that provide the heights of the barriers between attractor states (Figure 4) leading to the optimum cell state dynamics. To sum up, the probabilities of the cell states transitions are the parameters of the genome’s fitness (on a cancer-relevant timescale).

From the cancer genome’s perspective, the fittest (optimum) genome produces the cell state abundances which maximize, in the respective microenvironment on the cancer relevant timescale, the genome’s clone size. Straightforwardly, as the cell state abundances derive from the respective cell state stabilities provided by the genome-encoded epigenetic landscape [78], the optimum genome provides the landscape that generates, after the cancer-relevant time period, the largest clone. It implicitly presumes that the genome ensures the survival of its clone on all timescales shorter than the cancer-relevant one [104], which makes, due to inevitable fitness trade-offs between its ancestral genomes in direct line, the genome’s fitness very difficult to predict.

Regarding differences between respective epigenetic landscapes of normal and cancer genomes, the former evolved during the evolution of a (multicellular) organism, while the latter evolved during the organism’s life. As the unit of replication is the genome, the respective repertoire of the stable cell states (the cell states’ heterogeneity) provided by its respective epigenetic landscape undergoes selection as a whole. During long term evolution of metazoan body in the environment with specific spatiotemporal dynamics, the repertoire of stable cell states converged towards the optimum (from the metazoan perspective) repertoire, hence the optimum cell states abundances. In a changed environment, however, a different repertoire of stable cell states can be the optimum and the genome which finds (and exploits) it the most efficiently is, by definition, the winner of the evolutionary race. Two mechanisms of instabilities that provide the cancer genome with higher explorative capability were observed, (i) increased genetic instability, termed mutator phenotype [105], and (ii) increased epigenetic plasticity owing to more permissive epigenetic landscape [106]. To overcome the selection barrier and evolve cancer, mutated instability genes must provide neither too low nor too high genetic instability [107]. Similarly, the walls separating the cell states in the epigenetic landscape are lowered in premalignant or malignant cells (Figure 4), which enables the cancer genome to sample alternative transcriptional states and gene pathways [106].

To sum up, owing to higher genetic instability and more permissive epigenetic landscape, the cancer genome outperforms the normal genome in its capability to explore and exploit opportunities provided by the time varying microenvironment.

## 4. Cancer Clone as Cooperating Group

As isogenic cells in different states differ in their properties, non-genetic heterogeneity defines the complex interaction between cells, thereby it becomes the genome’s important evolutionary trait on the cancer relevant timescale. If the interaction increases the fitness of a multicellular body, it is usually termed as cooperation, which is typically the case of normal cells. On the other hand, cancer cells are often said to be selfish, regarding their impact on the metazoan fate. In the conceptual model of group selection [108], the groups composed of cooperators grow faster than the groups of defectors, whereas inside any mixed group, defectors reproduce faster than cooperators, which means that while selection at the lower (individual) level favours defectors, selection on the higher (group) level favours groups consisting of cooperators. It implies that, being the evolutionary winner at the selection level between the metazoan and single cell selection levels, the group of cancer cells forms the cooperating group. Intuitively, the interaction between the cells within an isogenic clone brings more parameters to maximize the genome’s fitness.

The question arises, what does the cooperation between the cells in isogenic cancer clone consist in—which is challenging research topic [109]. It was proposed, amongst other examples, that genetically distinct tumour cells cooperate to overcome host defences by exchanging different diffusible products [110,111], which indicates a specific evolving ecosystem. Some authors even suggest that tumours should be viewed as abnormal organs composed of multiple cell types and extracellular matrix, progressing through the processes that resemble development of organs and tissue remodeling [112]. In a few papers, the cooperative interaction between cancer cells is interpreted as an evolutionary public goods game, where the product (e.g., diffusible factors) is produced only by some individuals of the group (which pay evolutionary cost), but it is shared by all group members [113,114]. Intuitively, not only unequally produced (and equally shared) metabolites can play the role of public goods, but it can be any feature that decreases the fitness of the individual that provides it, and, at the same time, increases the fitness of those individuals which do not.

### 4.1. Exploration vs. Exploitation Dilemma

It often happens in real-world optimization problems that, due to their complexity, the only possibility of finding the optimum solution (or, at least, as good as possible one) is evaluating, one by one, the ‘candidate’ solutions. By intuition, the more candidate solutions are evaluated, the better result can be expected. However, as each fitness evaluation requires finite evaluation time and non-zero resources, the number of evaluations is, unavoidably, limited. The question follows how to sample the search space to maximize the probability of finding the optimum (or, at least, as good as possible) solution. As uncertainty of the stationary search space decreases proportionally to the number of already realized evaluations, rational strategy is to balance exploitation (i.e., reproducing the best of evaluated solutions) with the exploration (evaluation of not yet evaluated solutions), reducing during the search explorative component. However, the question immediately follows how fast the exploration to exploitation ratio should decrease. The problem is known as the exploration vs. exploitation dilemma [19] and optimization techniques differ in the way with how they solve it, with the efficiency crucially depending on the dimensionality, ruggedness, modality, stationarity, etc., of the search space.

The exploration vs. exploitation dilemma is especially challenging in the time-varying search space, as therein highly fit solutions can appear (at least temporarily) in not evaluated areas of the search space and, in reverse, less profitable (at that time) areas can be unproportionally often evaluated. As emphasized in the evolutionary optimization in dynamic environments [115], to benefit from the new opportunity, the evolutionary algorithm has (i) to detect change in the fitness landscape and (ii) efficiently respond to that change. To satisfy these requirements, in human-driven evolutionary optimization in dynamic environments, one purposefully allocates trials to tune the balance between exploration and exploitation. It is usually done by implementing the plethora of overcrowding mechanisms into the optimization procedure to penalize solutions that are too close (similar) each other. Alternatively, the subset of solutions is ad hoc chosen upon initialization and repeatedly evaluated [115,116]. These fixed solutions are chosen not regarding their instantaneous fitness but to cover optimally (in a statistical sense) the search space, thereby maintain the exploratory power of the algorithm. The maximum entropy principle states that if the probability distribution of a random variable is not known, the probability distribution which best represents the current state of knowledge is the one with the largest information theoretical entropy.

Regarding cancer-relevant timescale, time variability of a tumour microenvironment due to external factors (including therapy) and the cell state dynamics within the clone, come into play. It can happen that a microenvironment change provides new, more gainful cell states. To detect and, eventually, to benefit from that change, it must be ensured that enough cells are already in some of these cell states despite their low previous gains. If the cell is permanently ‘pinned’ to the specific state, its contribution to the genome’s fitness reflects instantaneous favourability or adversity of that state, which can be, due to the microenvironment’s time-variability, temporary. Therefore, even if stuck in, from the cell’s viewpoint, unfavourable state, the cell can increase the genome’s fitness by being ready to reproduce as soon as the adversity of the microenvironment vanishes. In this sense, the cancer cell contributes to the genome’s fitness not only by its reproduction gain, but it implicitly serves as a ’sensor’, monitoring the respective area of the cell state space. The latter capability, however, contributes to the genome’s fitness only if the long-term (cancer relevant) timescale is assumed.

The above conceptualization can be personified by a straightforward metaphor with the line of skirmishers searching through some area. More than on the capabilities of individual skirmishers, success of the group depends on how reasonably are the skirmishers coordinated in the area (and, eventually, time). Simply said, the output of the search cannot be attributed (at least not exclusively) to the superior capabilities of a single skirmisher (or a few of them), but rather to their coordination, which comes from the higher organization level. In the same way as the skirmishers are subordinated to the central level of command, the (cancer) cell states abundances are subordinated to the genome, which ‘exercises its power’ through the unique epigenetic landscape. What distribution of the genome’s instances (cells) in the cell state space maximizes its fitness on the cancer relevant scales depends on the spatiotemporal properties of the cells’ microenvironment. In the following section, an analogy with the risk diversification strategy bet hedging known in the optimization of a fluctuating environment is outlined.

### 4.2. Is Cancer Hedging Its Bets?

In a time-varying environment, the fitness of the cancer genome on the cancer-relevant timescale depends on how well it solves the trade-off between the exploration of the cell state space and the exploitation of the obtained information. The optimum trade-off between the two capabilities is attained by adaptive diversification. Populations that face an uncertain future and/or environment evolve one of two fundamental population strategies of adapting to environmental uncertainty, (i) the generalist strategy, producing a constant phenotype reasonably fit in any relevant environment and (ii) the bet-hedging strategy, which generates non-genetic diversity of the cell states probabilistically [117,118,119]. Crucially, the bet hedging is realized as alternative expressions of the same selection unit, not as a form of genetic polymorphism [120].

One can find instructive analogy in portfolio management, where, to protect against fatal losses, the investor diversifies risk by dividing his budget into a few stocks (usually proportionally to their expected returns) instead of investing his whole budget into the most promising one. As the trends in the returns of the stocks change in time, the portfolio (or its part) is systematically restructured to increase positions to reflect the new perspectives of the stocks. Apart from the changes on the market, the optimum investment strategy must take into account not only the potential benefit of the restructured portfolio but the cost of this restructuring (transaction costs) as well. In analogy with the portfolio management, the cell state abundances represent the portions of the population’s reproductive effort [121], or ‘stocks’, using the market context, and the investor who applies the bet-hedging strategy is the genome.

In laboratory studies of yeast and bacteria, the rate of phenotypic switching evolves to reflect the frequency of environmental changes [88]. In their model of survival in changing environments, Kussell et al., demonstrated, that the optimal switching between normal and persister bacterial cells, characterized by slow growth and increased ability to survive antibiotic treatment, respectively, depended strongly on the frequency of environmental change and only weakly on the selective pressures of any given environment [91]. Because of formal similarity of the evolving cancer cell population with bacteria, viruses, or yeast, it has been recently proposed that the structure of intratumour heterogeneity is an evolutionary trait that evolves towards the maximum clonal fitness at the cancer-relevant timescale in a changing (or uncertain) environment and that its structure corresponds to the bet-hedging strategy [122,123,124,125,126], which has been recently put into a therapeutic context [127].

## 5. Conclusions

Exploitation of evolutionary principles to steer cancer progression towards drug sensitive cancer cells is in current cancer research challenging topic [5,6,128,129]. Despite accepting evolution as modus operandi of cancer long time ago, to interpret some aspects of cancer progression, such as ITH, is difficult from the most straightforward evolutionary view with the cancer cell as the principal selection unit. Facing significantly increased heterogeneity of cancer cells, the paradox arises: how do cancer cells select for mutations in instability genes that not only confer no direct growth advantage to the cell itself but might even carry significant growth disadvantage [107]? To incorporate the eco-evolutionary principles into cancer therapy [7], the basic algorithmic structure of evolution with clearly identified selection unit, relevant timescale and the respective fitness should be refined.

In the paper, different roles of the two levels of ITH in cancer evolution are accented—genetic, due to genetic instability, and non-genetic, resulting from phenotypic plasticity. While the former is the fundamental component of evolutionary machinery providing diversity for the subsequent selection, the latter is the component of the genome’s fitness on cancer-relevant timescale playing the crucial role in time-varying tumour microenvironment. The cancer genome evolves the epigenetic landscape, resulting in the optimum (from the cancer genome’s view) cell state abundances, hence non-genetic ITH. Because of their different roles in cancer evolution, the two levels of ITH should be addressed differently by the therapy [129].

From the viewpoint of evolutionary biology, phenotypic diversification is an adaptation to environmental uncertainty that enables species to survive environmental adversity. As a rule, the tumour microenvironment is variable at the cancer-relevant timescale (including an eventual therapeutic intervention), therefore cancer evolution follows universal rules of evolution in a changing environment [130]. One of the observed and well studied strategies of population diversification in changing environment is the bet-hedging strategy [120,121,131]. The relevance of this strategy in evolution of cancer necessitates determination of the optimum bet-hedging profile in a specific cancer case, which is a challenging task. Bound to the same evolving structure, the genome, the two components of phenotypic heterogeneity (genetic and non-genetic) interact in a complex way, which dramatically complicates their more rigorous analysis [131]. In this paper we reviewed the arguments in favour of such an interpretation. Having attributed cancer cell state dynamics to appropriate universal dynamics, such as bet hedging, one can exploit its universal features to influence cancer progression by modifying fitness landscapes in more systematic way.

Last but not least, regarding the ongoing debate on the origin of cancer (such as between the proponents of the somatic mutation theory (SMT) [132] on the one side and those of the tissue organization field theory (TOFT) [133] on the other side, as well as the atavistic theory by Davies and Lineweaver [134]), there are obstacles that limit the interpretation power of the approach presented here. Firstly, the molecular cause of cancer can vary. For example, hypermethylation of the CpG islands in the promoter regions of tumour-suppressor genes is a major event in the origin of some cancers, but not in others [135]. Secondly, the “origin” of cancer is very vaguely defined and the cancer phenotype is typically linked with specific features, the hallmarks of cancer [136]. As the conceptual basis of the approach presented here is the evolutionary optimization in changing environment, defining cancer (and its origin) by acquiring some special properties is not appropriate, as it is built on different concepts. Thirdly, the presented approach views carcinogenesis as the optimization problem; nevertheless, we actually do not know how far from the optima the cancer genome is—taking into account that fitness is, due to changing environment, dynamic, one should rather accept that the genome is all the time pursuing the (moving) optimum rather than reaching it, which enables mutation to increase the genome’s fitness in the instant environment (i.e., the fitness landscape).
